# Exertional Rhabdomyolysis Among Active Component Members of the U.S. Armed Forces, 2020–2024

**Published:** 2025-06-20

## Abstract

Exertional rhabdomyolysis is a pathologic muscle breakdown associated with strenuous physical activity. A largely preventable condition, it persists as an occupational hazard of military training and operations, especially in high heat environments among individuals pushing their endurance limits. A total of 464 cases of exertional rhabdomyolysis were identified in 2024, corresponding to a crude incidence rate of 35.9 cases per 100,000 person-years, and is the lowest case count recorded during the 2020-2024 period of surveillance. The 2024 case numbers demonstrated a 9.6% reduction from the peak rate in 2023, and the proportion of 39.7% hospitalized in 2024 represented a 4.0% decrease from 2023. In 2024, all services, excepting the Navy, showed declines in incidence rates, ranging from 2.0% in the Coast Guard to 29.1% in the Air Force, compared to 2023. Consistent with prior reports, subgroup-specific crude rates in 2024 were highest among men, those less than 20 years old, non-Hispanic Black service members, Marine Corps or Army members, and those in combat-specific or ‘other’ military occupations. Recruit trainees continued to experience the highest rates of exertional rhabdomyolysis in 2024, with a rate more than 13 times greater than officers and enlisted members.

Initiation of a high-intensity physical activity at unaccustomed intensity or duration, particularly under heat stress, increases the risk of exertional rhabdomyolysis. ^1^ A potentially serious condition, exertional rhabdomyolysis requires vigilance for early diagnosis and aggressive treatment to prevent serious consequences. Rhabdomyolysis is characterized by the breakdown of skeletal muscle cells and leakage of intracellular contents (e.g., myoglobin, sarcoplasmic proteins, electrolytes) into the extracellular fluid and the circulatory system. Myoglobin is toxic to the tubular cells of the kidney and can lead to renal failure.

Rhabdomyolysis severity ranges from asymptomatic or mild elevation of serum muscle enzyme levels to life-threatening emergencies, such as electrolyte imbalances, acute kidney failure, disseminated intravascular coagulation, compartment syndrome, cardiac arrhythmia, or liver dysfunction. ^1 - 4^ The characteristic triad of rhabdomyolysis symptoms are muscle pain, weakness, and red- to brown-colored urine, due to high levels of myoglobin, although over half of patients do not have all of these specific symptoms. ^5^

The standard diagnostic criteria for exertional rhabdomyolysis are elevated serum creatine phosphokinase (CPK) levels, indicating myonecrosis, usually defined as a CPK level of at least 5 times the upper limit of normal, following recent exercise. ^2 , 3 , 6^

The condition is most commonly identified among new recruits at recruit training and combat installations, during the first 90 days of basic training, ^7 , 8^ but it can be observed in athletes accustomed to intense training, ^9^ particularly when they extend themselves to the maximal limits of their physical endurance. ^10^ History of heat illness or prior heat stroke have also been described as significant risk factors for recruits who sustained rhabdomyolysis, ^8 , 11^ revealing the potential for co-morbid conditions.

**What are the new findings?**

During the 2020-2024 surveillance period, 2024 evinced the lowest incidence rate of exertional rhabdomyolysis, nearly 10% lower than the peak observed in 2023. The Air Force showed the largest reduction, with incidence rates decreasing approximately 30% from the previous year.

**What is the impact on readiness and force health protection?**

Exertional rhabdomyolysis is a serious threat to military members than can limit their service effectiveness and potentially predispose them to serious injury. Risk of developing exertional rhabdomyolysis can be reduced with prompt recognition of symptoms by commanders, informed by awareness of environmental conditions, cognizance of troop fitness levels, emphasis on graded preconditioning prior to more strenuous training, and adherence to recommended work and rest ratios with appropriate hydration schedules, especially in hot, humid weather.

*MSMR* annually summarizes the numbers, rates, trends, risk factors, and locations of exertional heat injury occurrences including exertional rhabdomyolysis. This report includes updated surveillance data from 2020 through 2024. Additional information about the definition, causes, and prevention of exertional rhabdomyolysis can be found in previous issues of *MSMR* . ^7^

## Methods

The surveillance period ranged from January 2020 through December 2024 and included all individuals who served in the active component of the Army, Navy, Air Force, Marine Corps, Space Force, or Coast Guard. Due to small numbers, Space Force members were included in the Air Force population. All data used to determine incident exertional rhabdomyolysis diagnoses were derived from records routinely maintained in the Defense Medical Surveillance System (DMSS). These records document both ambulatory encounters and hospitalizations of active component members of the U.S. Armed Forces in fixed military and civilian (if reimbursed through the Military Health System) hospitals and clinics worldwide.

A case of exertional rhabdomyolysis was defined as an individual with ICD-9 and ICD-10 diagnostic codes in any position indicating a hospitalization or outpatient medical encounter with either “rhabdomyolysis” or “myoglobinuria,” plus a diagnosis in any position of one of the following: “volume depletion (dehydration),” “effects of heat and light,” “effects of thirst (deprivation of water),” “exhaustion due to exposure,” or “exhaustion due to excessive exertion (overexertion)” **(Table 1)** . ^12^ Each individual could be considered an incident case of exertional rhabdomyolysis only once per calendar year.

**TABLE 1.** ICD-9 and ICD-10 Diagnostic Codes Used to Define a Case of Exertional Rhabdomyolysis

Primary condition	ICD-9	ICD-10
Rhabdomyolysis	728.88	M62.82
Myoglobinuria	791.3	R82.1
Associated conditions	ICD-9	ICD-10
Volume depletion (dehydration)	276.5 *	E86.0, E86.1, E86.9
Effects of heat and light	992.0-992.9	T67.0 * -T67.9 *
Effects of thirst (deprivation of water)	994.3	T73.1 *
Exhaustion due to exposure	994.4	T73.2 *
Exhaustion due to excessive exertion (overexertion)	994.5	T73.3 *

Abbreviations: ICD-9, International Classification of Diseases, 9th Revision; ICD-10, International Classification of Diseases, 10th Revision.

* Indicates that any subsequent digit or character is included.

Cases of rhabdomyolysis associated with trauma, intoxications, and adverse drug reactions were excluded. ^11^ For health surveillance purposes, recruit trainees were identified as active component members assigned to service-specific training locations during coincident, service-specific basic training periods. Recruit trainees were considered a separate enlisted service member category in exertional rhabdomyolysis summaries by military grade.

## Results

**FIGURE 1.**
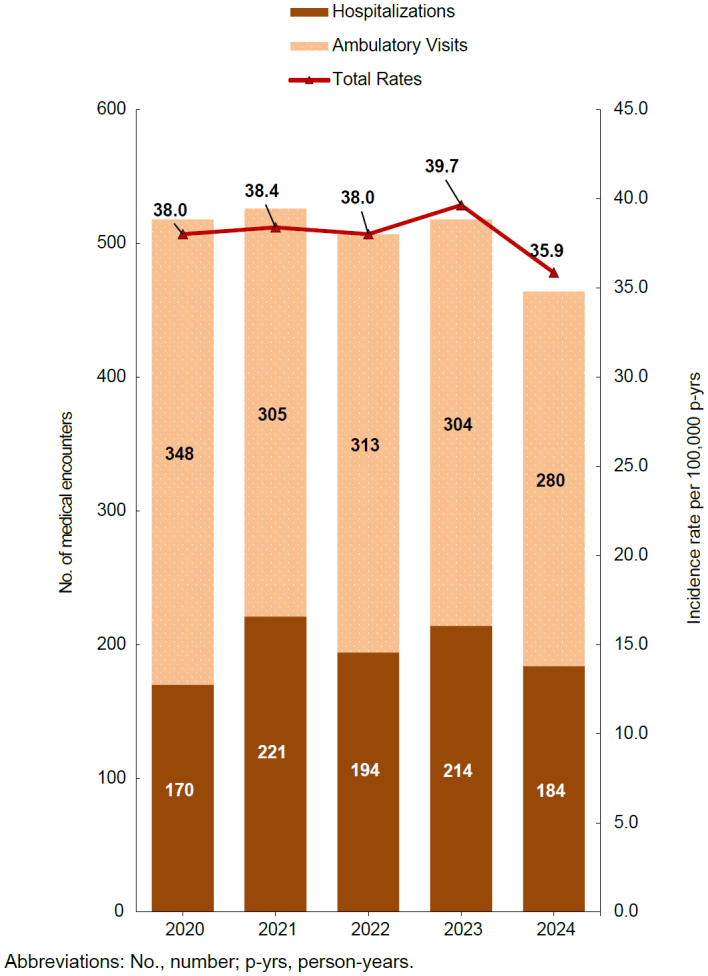
Incident Cases and Incidence Rates of Exertional Rhabdomyolysis by Source of Report and Year of Diagnosis, Active Component, U.S. Armed Forces, 2020–2024

In 2024, a total of 464 cases of rhabdomyolysis were identified that were likely associated with physical exertion or heat stress (i.e., exertional rhabdomyolysis)—which represents the lowest annual recorded number of cases during the 2020-2024 surveillance period **(Table 2** , **Figure 1)** . The crude incidence rate per 100,000 person-years (p-yrs) declined to 35.9, a 9.6% reduction from the peak rate of 39.7 observed in 2023 **(Figure 1)** . Incidence rates remained relatively stable, ranging from 38.0 to 38.4 cases per 100,000 p-yrs during the first 3 years of the surveillance period. The percent hospitalized was 39.7% (n=184), a 4.0% decrease from the corresponding percentage in 2023. Hospitalization proportions were lowest in 2020, at 32.8%, with an average of 38.8% over the 5-year period **(Figure 1)** .

**TABLE 2.** Incident Cases ^a^ and Incidence Rates ^b^ of Exertional Rhabdomyolysis, Active Component, U.S. Armed Forces, 2024

	Hospitalizations		Ambulatory Visits		Total	
	No.	Rate ^b^	No.	Rate ^b^	No.	Rate ^b^
Total	184	14.2	280	21.6	464	35.9
Sex	.	.	.	.	.	.
Male	171	16.1	258	24.3	429	40.3
Female	13	5.6	22	9.6	35	15.2
Age, y	.	.	.	.	.	.
<20	36	24.2	89	59.7	125	83.9
20–24	56	17.5	75	23.4	131	40.9
25–29	45	14.8	72	23.7	117	38.4
30–34	25	11.6	21	9.7	46	21.3
35–39	16	9.5	16	9.5	32	19.1
40+	6	4.4	7	5.1	13	9.6
Race and ethnicity		.	.	.	.	.
White, non-Hispanic	77	11.6	123	18.5	200	30.0
Black, non-Hispanic	50	23.8	72	34.3	122	58.1
Hispanic	44	17.0	57	22.1	101	39.1
Other / unknown ^c^	13	8.1	28	17.5	41	25.6
Service branch	.	.	.	.	.	.
Army	112	25.4	125	28.4	237	53.8
Navy	18	5.6	23	7.1	41	12.7
Air Force	15	4.7	23	7.2	38	11.8
Marine Corps	37	21.9	106	62.8	143	84.7
Coast Guard	2	5.1	3	7.6	5	12.6
Rank	.	.	.	.	.	.
Enlisted	133	12.9	178	17.3	311	30.2
Officer	29	12.0	29	12.0	58	23.9
Recruit	22	95.0	73	315.3	95	410.4
Military occupation	.	.	.	.	.	.
Combat-specific ^d^	49	30.3	63	39.0	112	69.3
Motor transport	3	7.1	8	19.0	11	26.1
Pilot / air crew	4	8.9	2	4.4	6	13.3
Repair / engineering	30	8.3	35	9.7	65	18.1
Communications / intelligence	40	14.4	43	15.5	83	29.9
Health care	10	9.5	11	10.4	21	19.9
Other	48	15.9	118	39.1	166	55.0
Home of record	.	.	.	.	.	.
Midwest	19	9.7	33	16.8	52	26.5
Northeast	31	20.0	42	27.1	73	47.1
South	86	15.2	137	24.3	223	39.5
West	36	12.0	55	18.4	91	30.4
Other / unknown	12	15.2	13	16.5	25	31.7

Abbreviations: No., number; y, years.

a One case per person per year.

b Rate per 100,000 person-years.

c Includes those of American Indian / Alaska Native, Asian / Pacific Islander, and unknown race or ethnicity.

d Infantry / artillery / combat engineering / armor.

Consistent with prior annual reports, crude incidence rates remained highest among men, those younger than age 20 years, non-Hispanic Black service members, Marine Corps or Army members, and those in ‘other’ and combat-specific occupations **(Table 2)** . Recruit trainees continued to have the highest rates of exertional rhabdomyolysis in 2024, at a rate of over 13 times greater than officers and enlisted members.

**FIGURE 2.**
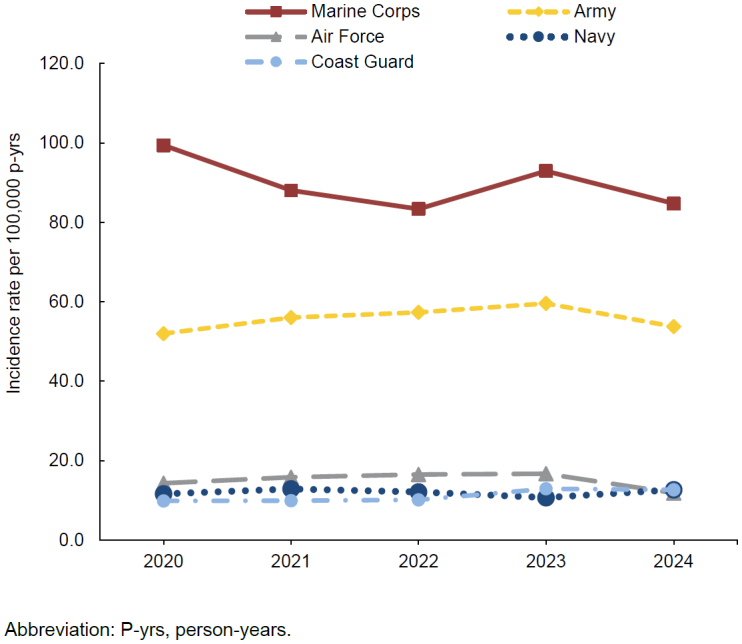
Annual Incidence Rates of Exertional Rhabdomyolysis by Service, Active Component, U.S. Armed Forces, 2020–2024

By service, the highest incidence rates of exertional rhabdomyolysis were observed in the Marine Corps and the Army, at 84.7 (n=143) and 53.8 (n=237) cases per 100,000 p-yrs, respectively. Rates in the Air Force (n=38), the Navy (n=41) and the Coast Guard (n=5) were similar, ranging from 11.8 to 12.7 cases per 100,000 p-yrs. With the exception of the Navy, all service branches showed declines in incidence in 2024, ranging from as little as 2.0% in the Coast Guard to as much as 29.1% in the Air Force, compared to 2023 **(Figure 2)** . The incidence rate of exertional rhabdomyolysis in the Navy was lowest in 2023 at 10.7 cases per 100,000 p-yrs but increased by 18.3% to 12.7 cases per 100,000 p-yrs in 2024. No cases were identified among Space Force members. When stratified by race and ethnicity, incidence rates declined the most among individuals of the other or unknown category, from 34.2 per 100,000 p-yrs in 2023 to 25.6 cases per 100,000 p-yrs in 2024, a 25.1% reduction (data not shown). The rate decreased by 16.7% among non-Hispanic White service members (from 36.0 cases per 100,000 p-yrs in 2023 to 30.0 cases per 100,000 p-yrs in 2024). Rates remained relatively stable among non-Hispanic Black and Hispanic individuals.

**FIGURE 3.**
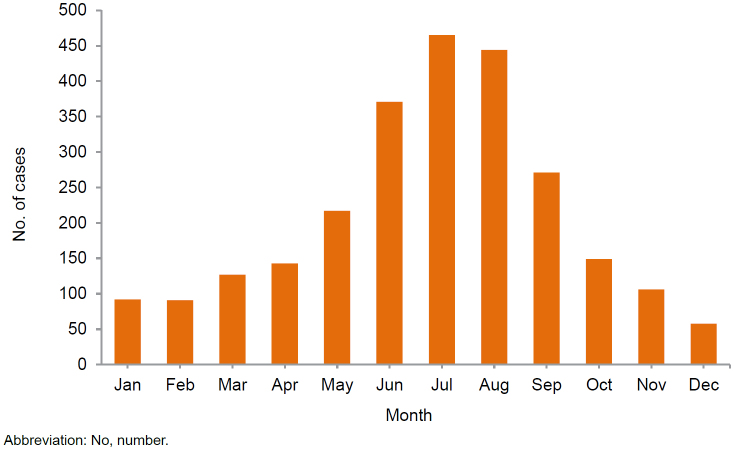
Cumulative Numbers of Exertional Rhabdomyolysis Cases by Month of Diagnosis, Active Component, U.S. Armed Forces, 2020–2024

During 2020-2024, approximately three-quarters (75.4%) of cases occurred during the warmer months (April through September) **(Figure 3)** .

During the 5-year surveillance period, 22 installations diagnosed at least 20 cases each; when combined, these installations diagnosed 67.6% of all cases **(Table 3)** . Of those 22 installations, 6 support recruit or basic combat training centers: Marine Corps Recruit Depot (MCRD) Parris Island/Beaufort, SC; Fort Benning, GA; Joint Base San Antonio-Lackland, TX; Fort Leonard Wood, MO; MCRD San Diego, CA; and Fort Jackson, SC; while 8 installations support large combat troop populations: Fort Bragg, NC; MCB Camp Lejeune, NC; Fort Campbell, KY; Fort Shafter, HI; Marine Corps Base (MCB) Camp Pendleton, CA; Fort Cavazos, TX; Fort Carson, CO; and MCAGCC Twentynine Palms, CA. From 2020 through 2024, MCRD Parris Island/Beaufort, Fort Benning, and Fort Bragg together accounted for over 1 quarter (26.8%) of all cases **(Table 3)** .

**TABLE 3.** Incident Cases of Exertional Rhabdomyolysis by Installation with at Least 20 Cases During the Period, Active Component, U.S. Armed Forces, 2020–2024

Location of Diagnosis	No.	% Total
Fort Bragg, NC	278	11.0
MCRD Parris Island, SC	211	8.3
Fort Benning, GA	191	7.5
NMC Camp Lejeune, NC	119	4.7
Fort Campbell, KY	109	4.3
Camp Pendleton, CA	85	3.4
Fort Cavazos, TX	79	3.1
Fort Shafter, HI	79	3.1
JBSA-Lackland, TX	72	2.8
Fort Leonard Wood, MO	60	2.4
MCB Quantico, VA	57	2.2
Fort Johnson, LA	48	1.9
Fort Bliss, TX	47	1.9
NMC San Diego, CA	38	1.5
Fort Carson, CO	37	1.5
Fort Jackson, SC	36	1.4
NH Twentynine Palms, CA	33	1.3
MCRD San Diego, CA	29	1.1
NH Beaufort, SC	29	1.1
NH Okinawa, Japan	28	1.1
Fort Belvoir, VA	26	1.0
Fort Eisenhower, GA	21	0.8
Other / unknown locations	822	32.4
Total	2,534	100

Abbreviations: No., number; MCRD, Marine Corps Recruit Depot; MCB, Marine Corps Base; NMC Naval Medical Center; JBSA, Joint Base San Antonio; NH, Naval Hospital.

## Discussion

This update documents that both the absolute case count and total crude incidence rate of exertional rhabdomyolysis declined in 2024, resulting in nearly 10% reduction from the peak incidence rate observed in 2023. The percent of cases that were hospitalized also declined by 4% but remained elevated compared to the lowest rate observed in 2020, which was likely influenced by COVID-19 pandemic restrictions.

This decline in the incidence of exertional rhabdomyolysis is encouraging. As a descriptive surveillance study, however, it cannot determine the specific reasons for the observed decline. It is possible that increased awareness following the 2023 peak may have led to enhanced prevention efforts, such as improved heat injury education, acclimatization protocols, and modified training regimens. Supporting the potential impact of training modifications, the recent incident involving the Tufts University men's lacrosse team, in which 24 out of 61 student athletes developed exertional rhabdomyolysis after being subjected to a high stress workout ^9^ demonstrates the importance of gradual acclimatization to allow adequate physiological adaptation to increasing demands of high intensity physical fitness training.

The Air Force experienced the largest decline among all service branches for exertional rhabdomyolysis incidence in 2024. The Navy was the only service in which no decline in incidence was observed in 2024. The Navy recorded its lowest rate in 2023, but in 2024 it rebounded to a level comparable to those observed during the first 3 years of the surveillance period, 2020-2022, suggesting that this increase may reflect a return to baseline or natural annual variation rather than a true increase in incidence.

Exertional rhabdomyolysis continues to occur most frequently from mid-spring through early fall at installations that support basic combat, recruit training, or major Army or Marine Corps combat units. Recruits can be exposed to environments requiring acclimatization to high heat or humidity in hotter months, while soldiers and marines in combat units often perform rigorous unit physical training, personal fitness training, and field training exercises regardless of weather conditions.

Non-Hispanic Black service members continue to have persistently higher incidence rates of exertional rhabdomyolysis, approximately twice the rates in other racial or ethnic groups. This observation has been attributed, at least in part, to an increased risk of exertional rhabdomyolysis among individuals with sickle cell trait (SCT), ^13 - 15^ for which the carrier frequency is approximated at 1 in 13 non-Hispanic Black individuals in the U.S. ^16 - 18^ The rhabdomyolysis-related deaths of 2 SCT-positive service members (an Air Force member and Navy recruit) in 2019 after physical training stress this potential risk. ^19 , 20^ Although studies had shown that SCT was associated with a 54% increase in exertional rhabdomyolysis risk, no similar association was found with risk of death. According to some experts, however, those studies missed deaths due to exertional sickling, and controversies with defining exertional rhabdomyolysis, its associations with disease progression and severity, prevention, and management evidence the need for further research. ^21 , 22^ Nevertheless, changes to the 2023 TRADOC Regulation include “sickle cell trait as a risk factor” as well as updated recommendations for screening, early recognition, and prevention of exercise collapse associated with sickle cell trait (ECAST). ^23^

The findings of this report should be interpreted with consideration of its limitations. A diagnosis of rhabdomyolysis alone does not indicate cause. Ascertaining the probable causes of exertional rhabdomyolysis cases was attempted by using a combination of ICD-9 / ICD-10 diagnostic codes related to rhabdomyolysis with additional codes indicating effects of exertion, heat, or dehydration. Other ICD-9 / ICD-10 codes were used to exclude cases of rhabdomyolysis that may have been secondary from trauma, intoxication, or adverse drug reactions. Recruit trainees were identified using an algorithm based on entry date into service, age, rank, loca tion, and time in service, which was only an approximation and likely resulted in some misclassification of recruit training status.

Management after treatment for exertional rhabdomyolysis, including the decision to return to physical activity and duty, is a persistent challenge for athletes and military members. ^21^ Service members who experience a clinically confirmed exertional rhabdomyolysis event should be further evaluated and risk-stratified for recurrence before return to activity or duty. ^6 , 24 , 25^ Service-specific guidelines may require temporary or permanent duty restriction following rhabdomyolysis, as recently diagnosed individuals remain at a higher risk for future heat illness. The most severe consequences of exertional rhabdomyolysis are preventable with effective mitigation measures and heightened suspicion of probability when environmental conditions favor muscular injury.

In 2024, the burden and incidence of exertional rhabdomyolysis was lower than in any other year of the 2020-2024 surveillance period, a nearly 10% reduction from the peak incidence rate observed in 2023. Continued surveillance will help determine whether these changes reflect sustained trends or temporary fluctuations. Service-specific public health and medical assets are encouraged to conduct targeted studies to identify and assess individual, operational, and environmental factors contributing to risk of exertional rhabdomyolysis, and evaluate the effectiveness of any preventive or mitigative interventions implemented. Commanders and supervisors at all levels should ensure that guidelines for heat illness prevention are consistently implemented, maintain vigilance for early signs of exertional heat injury, and intervene aggressively when exertional rhabdomyolysis is suspected.
